# INVESTIGATION OF BIOLOGICAL DETERMINANTS OF FRAILTY IN MALE AND FEMALE MICE

**DOI:** 10.1093/geroni/igac059.074

**Published:** 2022-12-20

**Authors:** Alice Kane, Patrick Griffin, Matthew Arnold, Maeve McNamara, Daniel Vera, David Vogel, David Sinclair

**Affiliations:** Institute for Systems Biology, Seattle, Washington, United States; Harvard Medical School - Blavatnik Institute, Boston, Massachusetts, United States; Harvard Medical School, Boston, Massachusetts, United States; Harvard Medical School - Blavatnik Institute, Boston, Massachusetts, United States; Volo Foundation, Jupiter, Florida, United States; Volo Foundation, Jupiter, Florida, United States; Harvard Medical School, Boston, Massachusetts, United States

## Abstract

Frailty is quantified as the accumulation of health deficits over an organism’s lifetime and gives a measure of the overall health of an organism. Preliminary studies have found an association between frailty and some of the hallmarks of aging including inflammation and senescence. However, the molecular underpinnings of frailty, and whether the mechanisms of frailty are distinct from or overlap those of aging is unknown. Previously we developed clocks based on frailty assessments that accurately predict age and lifespan in older male mice. Here, we expand these clocks to predict age, lifespan and frailty itself in younger, and also female mice. Additionally, we incorporate molecular measures including blood cell composition, plasma metabolomics, PBMC DNA methylation and stool microbial diversity measures into the clocks. These clocks provide important tools for the field, and also provide an indication of the molecular underpinnings of frailty and sex differences in these.

